# Thrombosis of Bilateral Profunda Femoris, Anterior Tibial, and Tibioperoneal Arteries in a Patient With COVID-19 Infection

**DOI:** 10.7759/cureus.17623

**Published:** 2021-08-31

**Authors:** Zeenat Khuda Bakhsh, Raheel Khan, Fatima Al-Khafaji, Vishnu Achyuth Suresh, Khalid Bashir

**Affiliations:** 1 Emergency Medicine, Hamad Medical Corporation, Doha, QAT; 2 Emergency Medicine, Hamad General Hospital, Doha, QAT; 3 Clinical Radiology, Hamad Medical Corporation, Doha, QAT; 4 Emergency Medicine, University of Buckingham Medical School, Buckingham, GBR; 5 Medicine, Qatar University, Doha, QAT

**Keywords:** hypercoagulability, thrombosis, covid 19, virchows triad, anticoagulation, arterial insufficiency

## Abstract

COVID-19, also known as severe acute respiratory distress syndrome coronavirus 2, mostly affects the respiratory system causing acute respiratory syndrome. It not only targets lungs but also causes vascular endothelial disruption, which can lead to arterial or venous thrombosis causing ischemia, which increases the morbidity and mortality in some patients, if not recognized and treated in a timely manner. We present an interesting case of a patient recovering from COVID-19 pneumonia , who developed bilateral foot ischemia due to thrombosis of bilateral profunda femoris, bilateral anterior tibial, and tibioperoneal arteries.

A 44-year-old gentleman presented to the emergency department complaining of severe bilateral foot pain, which progressively got worse. Upon examination he had blue toes bilaterally with absent dorsalis pedis and posterior tibial pulse. CT angiogram was performed, which showed severe multilevel lower limb arterial occlusions involving bilateral profunda femoris, bilateral anterior tibial, and tibioperoneal arteries. The patient was initially thrombolyzed and later underwent thrombectomy with the assistance of interventional radiologist. Hospital course was uneventful, and the patient was discharged on warfarin following complete resolution of symptoms.

## Introduction

Acute limb ischemia is a rare and potentially serious medical emergency. It is mainly due to sudden decrease in limb perfusion compromising limb viability [1] . Acute arterial occlusion occurs because of thrombosis of a diseased atherosclerotic artery, acute thrombosis of a stent or graft, dissection of an artery, hypercoagulablility, direct trauma, or due to an embolus from a proximal source dislodging into a distal vessel

Novel coronavirus, severe acute respiratory distress syndrome coronavirus 2, causing COVID-19 infection, results in an exaggerated inflammatory response leading to serious manifestations such as adult respiratory syndrome, sepsis, coagulopathy, and death [2]. Patients infected with COVID-19 infection are at high risk of thrombosis, which can be explained by Virchow’s triad due to abnormalities in the vascular endothelium, altered blood flow, and platelet dysfunction, causing arterial and venous thrombosis [3].

Diagnosis can be made with the help of bedside clinical examination, which may reveal skin discoloration of foot, delayed capillary refill time, and absent pulses. Doppler Ultrasound may show thrombus with absent signal or computerized tomography (CT) angiogram can show filling defect in the vessels due to thrombosis.

The management of acute lower extremity ischemia in the emergency department starts with providing adequate analgesics, stabilization of patient, anticoagulation with intravenous heparin followed by mainly surgical or catheter-based embolectomy and thrombolytic therapy or percutaneous transluminal angioplasty [4].

Early recognition and initiation of treatment reduces the complications and helps to provide the best chance for limb salvage.

## Case presentation

**Figure 1:**
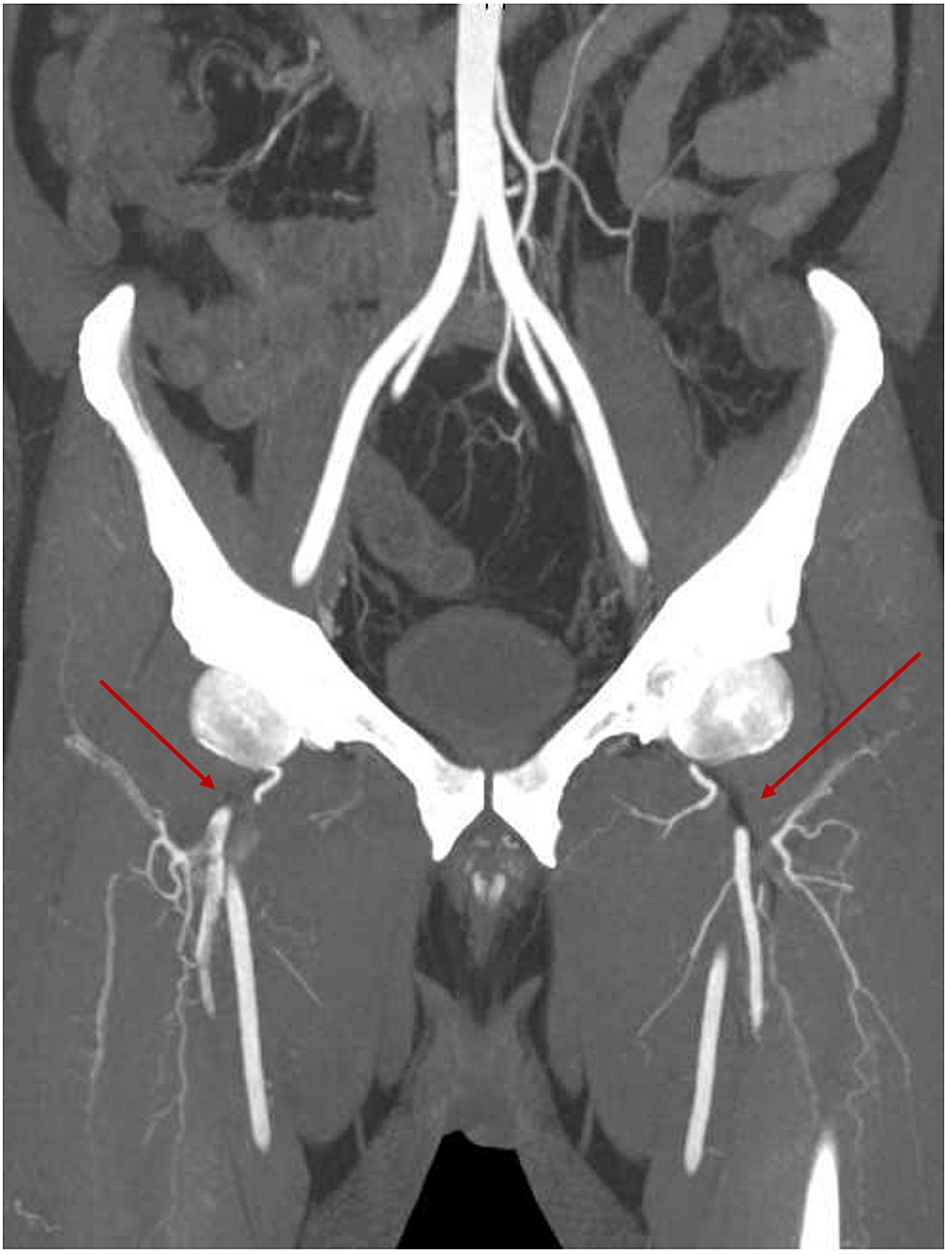
Computed tomography aortogram maximum-intensity projection showing filling defect within the profunda femoris artery bilaterally with a faint rim of contrast surrounding starting just distal to its origin and extending inferiorly as it gets its perforator branches

**Figure 2:**
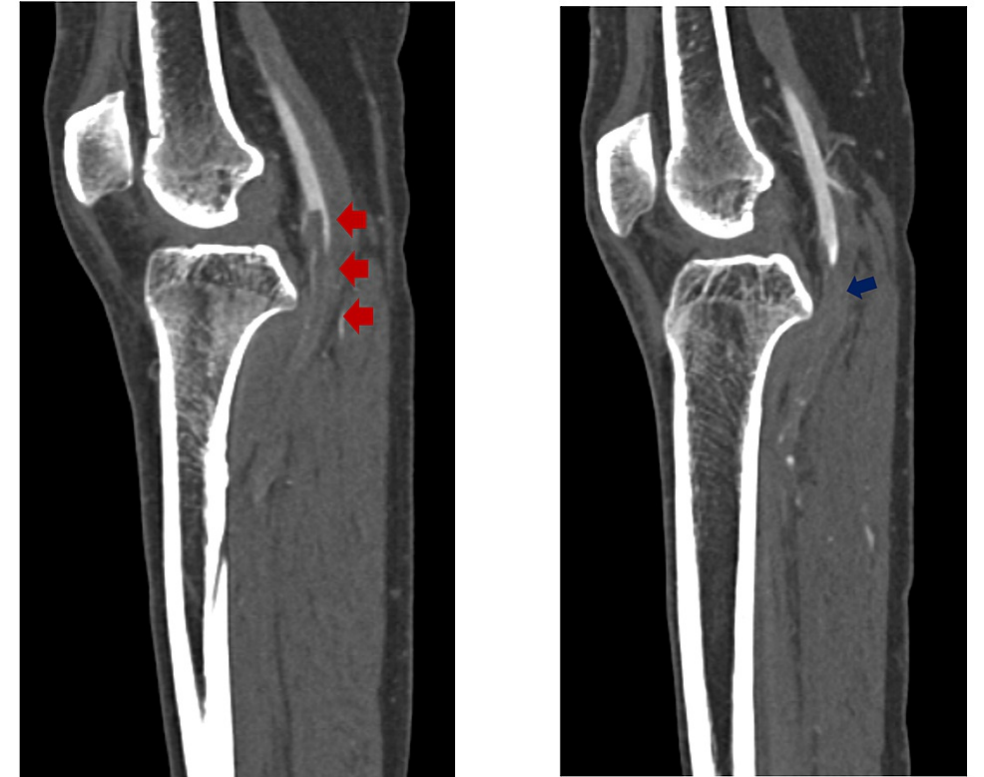
These are sagittal computed tomography angiography of both lower limbs, demonstrating complete occlusion of the right popliteal artery with downward thrombus extension to the anterior tibial artery (red arrow). Complete occlusion of the left popliteal artery thrombus with also downward extension of the thrombus to the anterior tibial artery (blue arrow)

**Figure 3:**
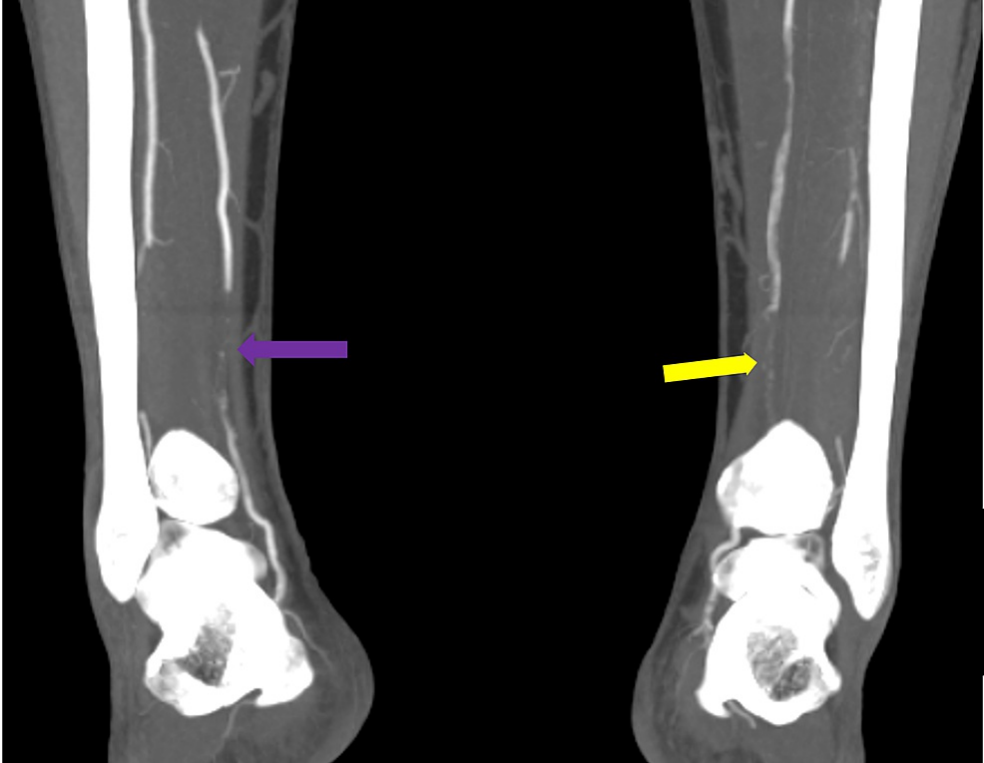
Maximum-intensity projection images of the lower limb angiography demonstrating multiple segmental thromboses in the right posterior tibial artery (purple arrow) and left posterior tibial artery (yellow arrow)

A 44-year-old male with no chronic illness, non-smoker presented to the emergency department complaining of severe bilateral foot pain for five days. The patient was discharged from COVID-19 quarantine facility three days before presenting to the emergency department after recovering from COVID-19 pneumonia. The patient had moderate severity of pneumonia requiring four days of hospitalization after which he stayed in quarantine facility for 14 days. The patient started to have bilateral foot pain, which progressively got worse, so he attended the emergency department. Upon arrival, the patient had active severe bilateral foot pain with numerical rating scale of 8/10 with bluish discoloration of both big toes. Upon examination he was afebrile and his vitals were stable. Foot examination showed bluish discoloration of both big toes, cold and tender foot distally with absent dorsalis pedis and posterior tibial pulses on both sides; however, bilateral femoral pulses were strongly palpable. Bedside ultrasound showed normal abdominal aorta from epigastrium to bifurcation with no thrombosis. The patient was taken for CT angiogram of both lower limbs, which revealed severe multilevel lower limb arterial thrombosis/occlusions involving bilateral profunda femoris (Figure 1), bilateral anterior tibial, and tibioperoneal arteries (Figures 2, 3).

Blood workup showed white blood cells 11.2 x 10^3^/uL with absolute neutrophil count 9.8 x 10^3^/Ul (N 2-7), prothrombin time 11.9 s (N 9.7-11.8 ), international normalized ratio (INR) 1.1, D dimer >35.20 mg/L fibrinogen-equivalent units (N 0.00-0.4), fibrinogen 5.91 g/L (N 1.70-4.20), and activated partial thromboplastin time 22.9 s (N 24.6-31.2). C-reactive protein was 103.5 mg/L (N 0-5) with normal electrolytes and renal parameters. The patient was heparinized using unfractionated heparin 25,000 unit(s) (14.7 unit/kg/h) infusion titrating according to the target of 1-1.5 times normal partial thromboplastin time and thrombolyzed using alteplase 2.5 mg bolus in both limbs via femoral arterial sheath. The patient was sent to Intervention Radiology suite for thrombectomy the same day. After procedure he was transferred to intensive care unit (ICU) where he continued alteplase infusion of 1 mg/h in bilateral lower limbs via femoral arterial sheath for 24 hours. Heparin infusion was continued during thromobolysis. The patient was bridged to warfarin 5 mg daily after five days targeting INR 2-3. Hospital course was uneventful, and he was discharged home on warfarin following complete recovery.

The patient was followed up in the outpatient clinic after two weeks, and examination showed bilateral pink-looking feet with about 1 cm of residual bluish discoloration of the big toes. Bilaterally pulses were felt in femoral and popliteal arteries, and positive biphasic Doppler signals were observed in posterior tibial, dorsalis pedis, and peroneal arteries. Capillary refill time was within normal range and the sensation was unremarkable.

## Discussion

We presented a case of bilateral profunda femoris, bilateral anterior tibial, and tibioperoneal arteries' thrombosis in a patient recovering from COVID-19 infection. It is rare to see bilateral distal foot arterial thrombosis in patients without prior vascular risk factors.

Acute arterial occlusion causing lower-extremity ischemia is a potentially serious diagnosis, which requires prompt recognition and treatment as mortality and limb loss rates remain high despite advancement in diagnostic and treatment modalities [5].

Arterial occlusion usually occurs in patients with atherosclerosis, injury to the artery by local trauma, hypercoagulability, or due to the dislodgement of thrombus from proximal arterial source. Certain diseases are prothrombotic, which can manifest as ischemic injuries. COVID -19 is now known to be prothrombotic [3].

In the beginning COVID-19 was mainly considered as a respiratory pathogen, but with passage of time, it appears to have caused multisystem involvement [6] including vascular complications.

Venous and arterial thromboses in COVID-19 can be explained by Virchow's triad [3], which consists of hypercoagulability , endothelial damage, and blood stasis. Virus can increase vasoconstrictor angiotensin II and decrease the vasodilator angiotensin, and the sepsis-induced release of cytokines can trigger a coagulopathy in COVID-19. Coagulopathy has been reported in up to 50% of patients with severe COVID-19 manifestations [4]. Prophylactic low-molecular-weight heparin has been recommended by the International Society on Thrombosis and Haemostasias and the American Society of Hematology [6].

In a study of COVID-19 patients requiring ICU stay, it was noted that the rate of thrombotic disorders in these patients was 31% [7]. Furthermore, images have shown that 27% of such thrombotic disorders are due to venous thromboembolism, 3.7% due to arterial thrombosis, and 81% due to pulmonary embolism [7].

## Conclusions

COVID-19 can affect multiorgan systems in addition to causing severe respiratory symptoms. It can cause arterial thrombosis due to various mechanisms like excessive inflammation, hypoxia, immobilization, and diffuse intravascular coagulopathy. It is rare to find limb ischemia in patients with COVID-19 infection. Prompt recognition and treatment can significantly reduce the complications and provide the best chance to salvage the limb.
